# Research and Technologies to Reduce Grain Postharvest Losses: A Review

**DOI:** 10.3390/foods13121875

**Published:** 2024-06-14

**Authors:** Bidhan Nath, Guangnan Chen, Cherie M. O’Sullivan, Dariush Zare

**Affiliations:** 1 School of Agriculture and Environmental Science, University of Southern Queensland, Toowoomba, QLD 4350, Australia; guangnan.chen@usq.edu.au; 2 Centre for Sustainable Agricultural Systems, University of Southern Queensland, Toowoomba, QLD 4350, Australia; 3 Senior Research Fellow, Tasmanian Institute of Agriculture, University of Tasmania, Launceston, TAS 7250, Australia; dariush.zare@utas.edu.au

**Keywords:** postharvest losses, grain, technology, supply chain, value losses

## Abstract

Reducing postharvest losses offers a significant opportunity to enhance food availability without requiring extra production resources. A substantial portion of cereal grain goes to waste annually due to a lack of science-based knowledge, unconscious handling practices, suboptimal technical efficiency, and inadequate infrastructure. This article extensively reviews losses occurring during postharvest operations across various crops, examining diverse postharvest operations in different countries. Recent advancements in postharvest technology research are thoroughly discussed. The primary obstacles and challenges hindering the adoption and implementation of postharvest technologies are also explored. The appropriate postharvest technology relies on specific factors, including the kind of crops, production locales, seasons, and existing environmental and socioeconomic conditions.

## 1. Introduction

Addressing the widening gap between food supply and demand within an increasing global population is a crucial concern for the development of humanity. Recent predictions suggest that the number of people worldwide will surge from around 7 billion to 9.7 billion by 2050 [1]. Most of this population growth will occur in developing nations [2]. This substantial increase, especially in urban and more affluent areas, will necessitate an approximately 70% boost in food production. Increased food demand can be attained through enhanced farm productivity or reduced waste. Numerous initiatives are underway to address the challenge of feeding an ever-growing population globally. Among these endeavors, a particularly noteworthy focus is minimizing postharvest losses. This specific measure clearly and positively correlates with safeguarding food supplies and conserving precious resources.

The term “postharvest loss/losses” (PHL) is concerned with the quantifiable losses in both the quantity and quality of food within the postharvest process [3]. PHL involves various activities, from harvest to eventual food consumption. It also involves the basics of food availability (consistent supply), food access (affordable purchasing power), and food utilization (proper storage and preparation).

Research on PHL significantly contributes to enhancing food security through offering sustainable strategies to improve food production and reduce losses and waste [4]. Overall, PHL reduction is often less expensive than an equal increase in food production and can result in greater returns compared to ramping up crop production [5]. As part of the Sustainable Development Goals (SDG) agenda, the United Nations (UN) also set a target in 2015 to reduce global food waste and loss by half by the year 2030 [6].

Unfortunately, PHL research has not received the required attention as a critical issue in many countries. Globally, it was noted that less than 5% of agricultural research funding has been allocated towards this particular issue [7]. Therefore, to acquire a thorough grasp of the present situation of PHL, a comprehensive review is required, accompanied by recommendations. This review assesses the existing literature on PHL, its underlying causes, the technologies in use, and potential measures for reduction. Specifically, this paper offers an in-depth analysis and discussion regarding the PHL situation in major cereal crops, the primary factors causing these losses, and a practical path forward. It also discusses concerns regarding on-farm and off-farm PHL. Finally, the social and environmental impacts of PHL are explored.

## 2. Grain Supply Chain and Postharvest Loss

Cereal grains have been essential in advancing human civilization and have constituted the fundamental element of the human diet for millennia. Wheat, rice, and maize, accounting for 89% of overall production, are the three most widely consumed cereal crops globally. Conversely, rye, oats, barley, millet, and sorghum remain a minority proportion of grain production.

**Figure 1:**
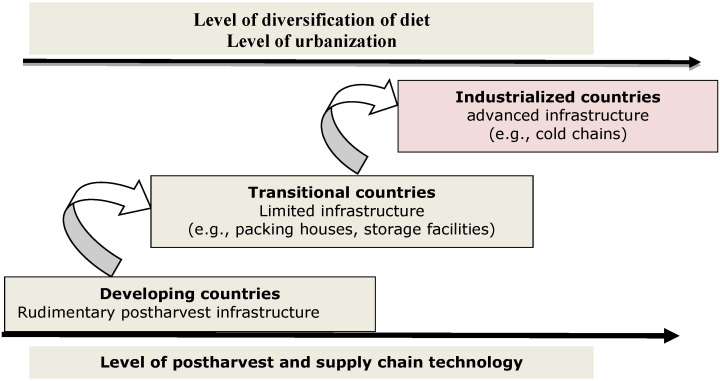
Illustration depicting the postharvest supply chain [12].

The postharvest supply chain involves a collective effort on and off the farm [8]. The on-farm system concerns harvesting, threshing, cleaning, drying, storage, transportation, hulling, packaging, and transporting to a market. Inversely, off-farm activities mainly include meal preparation, consumption, and infrastructure elements, such as roads, transport, warehouses, and marketing systems (Figure 1). Laskowski et al. [9] report that over half of the global daily caloric intake is derived from directly consuming cereal grains. Kumar and Kalita [10] further suggest that preserving cereal crops is the most effective way to fulfil food requirements and minimize economic pressure.

In developed countries, food loss is frequently observed in the stages very close to consumption, whereas in developing countries, it predominantly occurs at earlier stages, near the farm, such as processing, storage, warehousing, and logistics). In industrialized nations, advanced technologies and more efficient crop-handling systems in the middle of the supply chain contribute to a relatively low level of food loss compared to less-developed nations [11].

## 3. Extent of Postharvest Loss/Losses (PHL)

Postharvest losses (PHLs) can be measured in two ways: qualitatively and quantitatively [13]. Quantity loss refers to the decrease in weight or volume, while quality loss is measured by changes in the food’s physical condition, characteristics, and value. Food that becomes unsuitable for human consumption and is subsequently rejected is a key aspect of PHL [14]. Quantity losses are often more noticeable, especially in developing countries. Different agricultural products experience varying levels of loss: cereals have about 19% loss by weight, root crops around 20%, and fruits and vegetables about 44% [15].

Postharvest losses can be either physical or value-based. Physical factors like infestation and poor handling are often the most significant [16]. There is growing recognition of value-based losses, which consider quality and nutrition. These losses are influenced by government policies, consumer preferences, and trading strategies. For example, 53% of the caloric content in cereal crops may be lost [10]. Quality loss affects calorie content and edibility, which is more common in developed countries. Physical damage and quality losses reduce the economic value of crops, and in some cases, these losses can be as high as 80% of the total production [17].

## 4. Causes of Post-Harvest Loss

The primary causes of postharvest losses involve the varieties of crops and prevailing seasons. As a product progresses through the supply chain, PHL can result from various causes, such as climatic, genetic, and environmental factors. Many researchers have categorized the factors into two primary categories: internal and external. The key aspects of internal factors are gathering, separating, moving, drying or cooling, storing, evaluating, packaging, and labeling. On the other hand, external factors are typically associated with environmental conditions such as temperature, humidity, and socioeconomic patterns [18].

### 4.1. Genetic Variation

Postharvest losses in cereal grains are relatively low; however, certain crop varieties are inherently more susceptible to losses than others [14]. For instance, wheat, maize, and barley cultivated during the dry season tend to be more resilient to PHL than rice. In comparison, hybrid varieties with more grains in the panicle generally experience higher loss levels than inbred varieties.

### 4.2. Climatic Conditions

Postharvest activity depends upon climatic conditions, especially temperature, humidity, and rain intensity [19]. It is often recommended to avoid cloudy weather (maintaining relative humidity below 70%) when storing cereal crops to prevent mold development in the grains [20]. While rainfall is advantageous during a crop’s growth phase, it can pose significant problems during harvesting seasons, increasing wastage rates. Therefore, ongoing efforts to mitigate losses caused by weather-related factors are essential for sustaining optimum environmental conditions.

### 4.3. Maturity of Grain and Postharvest Operations

The maturity and ripeness stages of crops also impact the quality and quantity of postharvest losses [21]. Each crop type has a specific lifecycle or harvesting window, which depends on its psychological characteristics, local climate, and growing region. For instance, winter and summer wheat and rice crops exhibit distinct lifespans, and this variance directly impacts PHL. Moisture content and grain color are useful for determining the timing of postharvest operations, including harvesting, drying, storage, etc., thereby aiding in reducing PHL (Table 1). Achieving optimum maturity (for rice, 25% MC or 80% of grains becoming straw color) can significantly mitigate losses.

However, the timing of grain harvesting is often strongly influenced by both market demand and the availability of storage facilities. In some areas of Asia and Africa, growers might harvest their crops before they reach full maturity due to immediate financial needs or food shortages. This practice can lead to a decrease in both the nutritional content and economic worth of the crops. Additionally, selected farmers may choose to harvest their crops before they fully mature to fetch higher prices in the market. Ultimately, it is up to the growers to decide when and how to harvest to maximize their gains and minimize losses. The scope of PHL depends on the choices made by these farmers.

## 5. Main Cereal Crops and PHL

Among the essential crop grains, cereals experience the highest proportion of postharvest losses [22]. Hence, postharvest intervention over the whole food supply chain reduces PHL. Despite this, reliable and accessible postharvest data are still limited. In the following sections, we will examine the existing literature regarding the impact of postharvest loss (PHL) from currently accessible technology.

### 5.1. Rice

Rice stands as the predominant staple food for a significant portion of the global human population, particularly in Asia, where an overwhelming 90% of the world’s rice is grown [23]. Regarding production, rice is the third among global agricultural commodities, with a production volume of 776.5 million tonnes. It follows sugarcane, which has an overwhelming 1.9 billion tonnes, and maize, which accounts for 1.24 billion tonnes (www.statista.com/statistics/263977/world-grain-production-by-type, 2023/24, accessed on 15 May 2024). Approximately 3.5 billion people across the globe depend on rice as their primary source of food, contributing 20% of the calories in nations with lower to middle incomes [24]. Farooq et al. [25] recently estimated that the global need for rice will persist upward, with projections indicating an increase from 479 million tonnes in 2014 to approximately 536–551 million tonnes by 2030, driven by anticipated population growth. Moreover, rice contributes to one-fifth of the global calorie supply [22].

**Figure 2:**
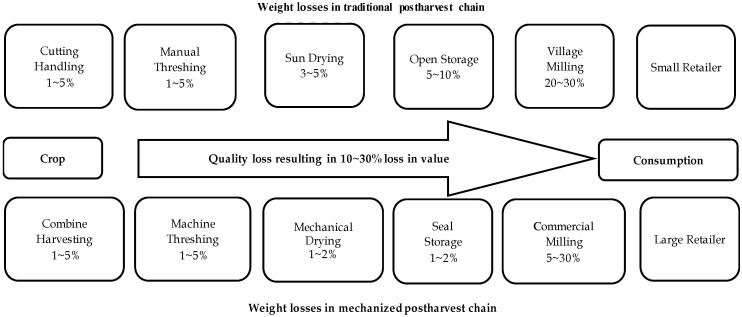
Estimated losses (weight and quantity) along the postharvest chain for rice in South Asia [39].

Postharvest losses present a substantial challenge across the entire rice and food supply chain. Rice in South Asia suffers significant losses throughout the postharvest chain, impacting both weight and quantity. Figure 2 illustrates the traditional and mechanized postharvest operation chain, revealing significant weight, quantity, and quality losses from crop production to consumption. Estimates suggest these losses can range from 10% to 30%, leading to a substantial decrease in the final value of the rice. Various factors, including a country’s economic conditions, geographical locations, technological practices, and climatic conditions, further influence the supply chain, as detailed in Table 2. In many developing nations, particularly South Asia and Africa, physical losses in rice production can range from 10% to 30%, highlighting the need for improved postharvest management practices.

According to estimates from the World Bank in 2010, PHL for rice in India ranged from 7% to 10% at the farm level, while at the market and delivery levels, it varied from 4% to 5% [26]. Another study [27] reported that the PHL of rice varied from a minimum of 3.51% in India to a maximum of 24.9% in Nigeria. In comparison, the total rice losses in the entire supply chain, from producer to retailer, were approximately 10.74% and 11.71% in Bangladesh [5]. This loss is broken down as 10.74% for the Aman season, 11.71% for Boro, and 11.59% for the Aus rice growing periods.

Quality losses can reduce prices by up to 30% [14]. The PHL of grain across Asian countries is usually estimated to be between 10% and 15%, potentially decreasing its value by 25% to 50% [28]. According to Alizadeh and Allameh [29], between 85.28% and 87.77% of the total PHL of rice takes place during farm-level operations, with storage-related losses being a significant factor, accounting for the most considerable portion (between 33.92% and 40.99% at the farm level).

Interventions in the PHL reduction strategy (both for production and marketing) are designed to save the crops that are produced. Kiaya [14] noted that significant resources have been spent worldwide to reduce rice PHL, most of which have concentrated on farm-level losses. Alternatively, to minimize rice PHL, a new concept, the “Rice Processing Center (RPC)”, has been implemented in several countries, including Korea and Vietnam [30].

**Table 2:** Available information regarding postharvest losses of rice in different countries.

Country	Losses (%)	Significant Findings	References
India	6.21~7.34	Maximum losses during storage at field level	[31]
Bangladesh	10.74~11.71	Maximum losses during storage	[32]
China	8–26.0	Significant losses due to insect infestation during storage	[10]
El Salvador	6.0	-	Unknown
Indonesia	12.0	These losses are from farm-level activities	[33]
Nepal	15.0	Significant losses at the small-scale farm level	-
Nigeria	24.9	-	[27]
Sri Lanka	12.5	-	[34]
Thailand	14.0	These losses are only from farm-level activities	[33]
Nigeria	24.9	Maximum losses during storage	[27]
Philippines	9~30.0	Maximum losses during drying	[35]
Malaysia	6.5	The highest losses are from farm-level activities	[36]
Pakistan	10.0	These losses are only from farm-level activities	[37]
Indonesia	12.2	Maximum losses during storage	[38]
Vietnam	7.0	These losses are only from the farm-level activities	[33]

### 5.2. Wheat

Wheat is a fundamental food source in various nations, including North America, Europe, Australia, and New Zealand. Compared to developed countries, developing nations face increased losses in wheat after harvest, particularly during harvesting operations [10]. Research by Alam et al. [32] indicated that in Bangladesh, wheat storage losses were most significant, comprising 41.7% of the overall losses after harvesting. Kumar Balai et al. [40] reported that the total loss within the wheat supply chain (from harvesting to retailing) amounted to 4.32% in Karnataka state, India. Sudan and Zimbabwe had an estimated wheat postharvest loss rate of 6~19% and 10%, respectively [41]. The high PHL during storage operations is primarily because of inadequate storage facilities.

### 5.3. Maize

Maize, known as corn in North America, has become a staple food in diverse regions globally, including Latin America, Sub-Saharan Africa, and Asia. Its total production exceeds that of rice and wheat. Maize is often allocated for uses beyond human consumption, such as animal feed, ethanol, starch, syrup, etc. Like wheat and rice, there are notable losses in the postharvest handling of maize in developing nations. 

Regardless of the scale of the farm, the majority of these losses occur due to insufficient technology and a need for more information [42]. In a household-level survey by Kaminski and Christiaensen [33] in three Sub-Saharan African countries (Uganda, Tanzania, and Malawi), farm-level PHL for maize crops ranged from 1.4% to 5.9%. The primary cause of these losses was damage from insects and pests during storage. The average value chain loss in ASEAN countries was 23%, with the most significant losses occurring during field drying (9%) [43]. Similarly, in Togo, there is a 0.2% to 11.8% loss in maize weight because of insect infestation, occurring after six months of storage, in conventional granaries [44]. A similar situation is observed in Guatemala, where postharvest losses were estimated at 40% to 45%, mainly due to inadequate storage structures and frequent unfavorable weather conditions [45]. In the Philippines, the crops are dried along the roadside due to a lack of suitable storage facilities, leading to significant losses.

## 6. Postharvest Losses in Non-Major Rice-Growing Developing Countries

However, in several developing nations that do not have significant rice production, rice, wheat, corn, and other crops continue to be cultivated to meet their food needs. For example, in East and Southern Africa regions, the average loss of grains due to PHL ranges from 10% to 20% in weight, with some areas experiencing even higher rates of 25% to 35% [46]. Focusing on specific crops, the PHL for rice in African countries is approximately 12.3%; for sorghum—it is around 11.6%; for wheat—it is 9.9%, and for maize—it is approximately 16.8% [34]. Additionally, Getnet and Kebede [47] reported that Korea experiences PHL rates of 15.56%, 16.65%, and 16.35% for rice, maize, and wheat, respectively. The data are summarized in Table 3. Inadequate technology is a key factor, contributing to elevated levels of PHL.

## 7. Global Postharvest Losses across Different Countries

The amount of rice lost after harvest (PHL) varies greatly depending on two main factors: how much rice is lost in the first place (severity) and how well a country can reduce those losses (mitigation). Different countries also have different levels of PHL [48]. Developed countries generally have lower PHL because they have a more organized system for growing, storing, and selling rice. This includes things like farmers working more closely with distributors and stores, stricter rules about how safe the rice must be, and using better technology throughout the process [28]. Australia, Europe, and the United States are some examples of countries with lower PHL. These countries have significantly reduced PHL by developing new technologies and ensuring that everyone in the rice industry has access and uses them.

In comparison, the uptake of postharvest technology has been slow in Africa, Asia, and Central America, resulting in significantly higher levels of PHL. In these countries, grain supply chains lack the basic postharvest infrastructure, rely on essential technologies, use outdated storage facilities, and need more connectivity to local and rural markets. Omotajo et al. [49] highlight the inadequacy of postharvest infrastructure as a chief contributor to PHL in numerous less-developed nations.

Sawaya [50] further revealed that high-income nations experience higher volumes of lost and wasted grain at the later supply chain stages (consumption levels). Conversely, low-income regions face the opposite situation, with more grain loss and waste occurring in the earlier stages.

### 7.1. Developing/Less Developed Countries

Limitations in finances, management, and technology are the main factors leading to grain losses and wastage in developing nations [14]. Despite considerable efforts in these nations to increase food production, a significant portion is still lost at the on-farm stage because of a deficiency of knowledge, information, technology, and national policies.

The UN Food and Agricultural Organization (2017) reported that an overall PHL rate of about 10~15% for grains is typical in less-developed nations. In India, approximately 23 million tons of cereal, 12 million tons of fruits, and 21 million tons of vegetables are lost annually. Similarly, in several African countries, 25% of the total harvested crop of cereal grain is estimated to be lost [51]. Additionally, PHL in Africa ranges widely from 20% to 40%, which is significant given the continent’s relatively low agricultural productivity [52].

### 7.2. Developed Countries

Developed countries often use advanced technologies and methodologies, resulting in significantly lower PHL throughout the supply chain, except at the point of consumption [10]. Additionally, industrialized nations possess extensive and efficient storage and cold chain systems, ensuring a prolonged shelf life for products [53]. Furthermore, well-organized farming systems, improved transportation, management, and upgraded storage and processing facilities lead to a greater proportion of harvested produce making it to the markets in developed nations. However, postharvest losses at consumption stages are still high in developed countries compared to less developed ones. As van Gogh et al. [54] highlighted, in developed nations, 23% of losses occur during the consumer stage, with the remaining 11% occurring at different points within the supply chain.

## 8. Postharvest System Elements, Impacts, Losses, and Mitigation Strategies: Cereal Grains

To effectively minimize PHL, it is crucial to understand and manage these contributing factors systematically. Table 4 concisely overviews potential triggers, elements, and factors contributing to cereal grain supply chain losses. While the listed steps are significant, additional factors may lead to postharvest losses.

### 8.1. Pre-Harvest Loss

Pre-harvest crop losses are reductions in yield during the growing season due to biological and environmental factors, primarily caused by insects, pests, diseases, and deterioration [55]. Extreme environmental events like floods, flash floods, cyclones, and droughts can occasionally lead to substantial pre-harvest losses [56]. Additionally, biological factors such as genetic predispositions to shattering and grain damage fall under the category of pre-harvest losses. The optimal selection and development of crop varieties are critical to mitigating these losses at the field level.

While high-yielding varieties can enhance productivity, they may also be more susceptible to rapid shattering, potentially resulting in postharvest losses that challenge farmers [57]. Affognon et al. [48] emphasized a need for studies linking concerns about developing new crop varieties to the reduction and impact of pre-harvest losses, whether in quantity or quality. Genetic engineering holds significant potential to reduce quantity losses and prevent or reduce vulnerability to mycotoxination contamination [16].

### 8.2. Harvest Loss

Harvesting is a crucial phase in the grain supply chain, involving the collection of mature grain from the field. This task is done manually in developing and less-developed countries, utilizing tools like sickles, knives, scythes, and cutters. Conversely, developed nations rely on combined harvesters or machinery for most of their harvesting needs [58]. The most significant determinants of harvest losses are the method of operation and the prevailing weather conditions (Table 5). In principle, hand harvesting allows for greater precision due to the detailed care it enables. However, labor availability constraints can result in delays or even failures to harvest on schedule. Nations such as India and Bangladesh have faced a labor deficit during peak harvesting seasons, resulting in significant losses. Moreover, Raut et al. [59] highlight that weather conditions during harvest are critical in postharvest losses (PHLs). Unforeseen weather events, like rain, can dampen the crop, encouraging mold growth and elevating the likelihood of aflatoxin or mycotoxin contamination.

#### Minimization of Harvest Loss

The implementation and wide adoption of suitable harvesting technology hold significant potential for minimizing PHL [16]. Mechanized harvesting emerges as a viable solution for farms of various scales, both large and small. In these contexts, adopting mechanized techniques, particularly the use of combine harvesters, presents an effective means to harvest cereal crops [60]. This not only results in reduced production costs but also enhances labor productivity. Consequently, many Asian countries have implemented measures to adopt advanced technologies that are compatible with their specific agricultural contexts. It is worth noting, however, that while combine harvesting can lead to reduced labor demands, there is a slight increase in grain loss (approximately 3%) [61].

### 8.3. Transportation Loss

Transportation is integral to moving grain from the farm to the consumer level. The various movements of the crop are the primary cause of high transportation losses [10]. The second most substantial expense is hiring labor for loading and unloading trucks. Due to inadequate road infrastructure and insufficient maintenance, transportation often leads to significant spillage and extensive contamination. In developed nations, on the other hand, losses during transportation are minimal due to better road infrastructure and well-equipped facilities. Conversely, in developing countries, the manual handling of loading and unloading grains from wagons, trucks, and rails at processing and storage facilities frequently leads to significant spillage.

Insufficient and inadequate transportation infrastructure often leads to damage to grain. In South Asian countries, a notable proportion of crops are transported directly from the field, utilizing bullock carts, head-carrying, or open trollies. Conversely, small-scale farmers engage bicycles, tractors, trailers, or taxis, contingent on available transport options and the volume of grain to be moved. Unfortunately, these transportation methods often lead to high PHL because the grain is usually poorly protected from pests, birds, contamination, and theft. For instance, in countries like India and Pakistan, bags of wheat are loaded and unloaded from vehicles as many as ten times before reaching the milling stage, with some grains potentially lost in each handling.

Moreover, the type of bag used for grain storage and transport plays a crucial role in minimizing waste. Substandard jute bags, susceptible to leakage, are frequently used for transportation and storage, leading to higher spoilage rates. The hooks that lift large bags (containing 80 to 100 kg of grain) may cause tears, resulting in increased spillage [62]. Kumar and Kalita [10] estimated rice transport losses in Southeast Asia and reported losses ranging from 2% to 10% during handling and transportation.

### 8.4. Threshing/Shelling Loss

Threshing is a physical procedure that involves the separation of grains from the surrounding straw, stems, and panicles and is a crucial step in agricultural practices [63]. This method can be achieved through various techniques, such as rubbing, stripping, or combining. In less mechanized regions, particularly in developing countries, threshing is predominantly manual, using methods like trampling and beating [64]. Small-scale farmers often rely on these manual approaches, though some may integrate machinery like maize shellers or threshers for more efficient operations. In contexts like rice postharvest handling, mechanical threshers are advantageous. Notably, in densely cropped areas of South Asian nations like Bangladesh, Nepal, and India, where grain losses are significant, there is a rising demand for mechanical threshers [46]. In contrast, industrialized nations have used combine harvesters for wheat, maize, and rice, as these machines encompass a range of tasks, including harvesting, threshing, and cleaning [65].

The method and timing of threshing are pivotal determinants of threshing losses. These losses can result from grain dispersion, seed spillage, inadequate separation of grain from chaff and straw, and potential grain breakage caused by excessive force during threshing [26]. Some crops, such as maize, millet, and sorghum, may be threshed months after harvest, and unprocessed crops stored in exposed cribs can lead to significant losses in quantity and quality. The primary drivers for delayed threshing are the lack of mechanization and these crops’ comparatively lower market value. Ahmad et al. [66] studied rice threshing methods and revealed significant impacts on weight and value loss, which are attributable to grain losses in muddy yards and damaged grains after milling.

Introducing diverse threshing technologies represents a significant stride towards minimizing losses arising from the separation and scattering of cereal grains. Adopting power threshers and combine harvesters empowers farmers to attain efficient and timely harvesting and threshing processes, outperforming conventional methods like beating and trampling [67]. Specialized machinery tailored for smaller-scale operations, such as maize shellers and rice-wheat threshers, has proven invaluable in developing nations. To reduce the risk of fermentation and spoilage, it is strongly advised to thresh rice on the same day as harvest. This proactive approach ensures the preservation of grain quality and maximizes the yield potential of the harvest.

### 8.5. Cleaning Loss

Cleaning is the intermediate operation between the drying and storage processes and is essential for enhancing value addition [68]. The process involves extracting damaged grains and foreign materials from whole grains like straw, stones, sand, chaff, and weed seeds. In developing countries, winnowing (using natural air) is a conventional method used for grain cleaning, achievable through manual or mechanical means [69,70]. This study highlights the critical importance of thorough and efficient cleaning processes in preserving harvested grains’ quality and market value.

### 8.6. Drying Loss

Drying is a significant and challenging phase in the grain production process. It involves reducing the moisture content of the grain to a level for safe storage [71]. The drying process can impact various attributes related to product quality, including sensory, nutritional, and functional aspects [72].

Drying loss depends on various factors, such as the chosen drying methods and equipment. Drying techniques can be natural (sun or shade) or mechanical (dryers). Sun drying, a traditional approach, is commonly used for drying harvested crops, including parboiled rice. It is a popular method in many developing countries [10]. According to Bala [71], the overall physical losses from the harvesting stage to sun drying were under 7%, while drying losses for wheat varied between 1.56% and 5%. In contrast, mechanical drying, gaining popularity among medium- to large-scale farms or commercial operations with substantial budgets, is seldom utilized by smallholders in developing countries [73].

While successful drying is essential, it cannot wholly prevent postharvest losses, as insects, rodents, and birds can still attack well-dried grain in the field before harvest or invade drying cribs and storage areas after harvest [74]. In less developed countries, when grains are left in open spaces for sun drying, they are susceptible to consumption by birds and insects. They may also become contaminated with materials like stones, dust, and other foreign matter. Farmers in Zambia and Zimbabwe utilized raised platforms to mitigate contamination, resulting in comparatively lower maize drying losses of 3.5% and 4.5%, respectively [27].

Moreover, the drying process depends on moisture levels and can lead to significant losses (Table 6). Grain with a moisture content of 18% to 26% or higher will deteriorate if drying does not commence within 24 hours of harvesting. Any delay in grain drying, whether incomplete, ineffective, or excessive, can degrade quality and lead to postharvest losses [10]. Over-drying can cause fissures in the seed and damage the embryo husking in a rice huller, impacting market value.

#### Minimization of Drying Loss

Ensuring the quality of produced grain through drying is critical at the on-farm level due to various abiotic factors, such as insects, pests, and climatic conditions. Both outdoor and indoor grain drying operations influence postharvest losses. The crop might be immediately transferred from the field to a cleaned, paved, or concrete surface at the homestead in specific areas. This drying process minimizes the likelihood of dust and foreign material contamination, reducing postharvest losses [76].

In contrast, utilizing power-driven drying methods presents several advantages over natural drying. These include decreased handling losses, enhanced control over hot air temperature, and more efficient utilization of space [72]. However, the high initial and maintenance costs and a lack of operational knowledge pose challenges for smallholders in utilizing mechanical dryers. As an alternative, solar energy-assisted dryers present a low-cost, simple design suitable for small- to medium-scale operations. Smaller-sized solar dryers in regions with hot, arid, or semi-arid climates have the potential for grain-drying [77]. In industrialized countries, technologies like NIR-based dryers, microwave-assisted dryers, and convective hot air dryers are used, ensuring both quality and market value despite their high installation costs [78,79,80,81].

### 8.7. Storage Loss

Storage serves the vital purpose of preserving the quality of agricultural products and preventing their deterioration beyond their typical shelf life. Several elements play a role in the decline of the quality and quantity of grains while in storage, with temperature and moisture content emerging as the most influential factors affecting attributes like seed germination, milling quality, grain color, and commercial value [82]. For example, ambient temperature ranging from 20 to 40 °C coupled with a relative humidity surpassing 70% establishes a conducive environment for the rapid growth of storage molds [83]. This climate also facilitates infestations by storage insects like *weevils*, *lesser grain borers*, and *khapra beetles*, particularly in the case of rice. Moreover, humid conditions and storage insects can worsen the production of aflatoxin.

Many studies [84,85,86] have demonstrated the key role of storage within the food supply chain. In developing nations like Bangladesh, Myanmar, and India, approximately 50~60% of grains are kept in conventional facilities at the farm and household levels, primarily intended for consumption and seeding, leading to notable losses. This figure is even higher in countries with challenging natural events and climatic conditions, such as frequent heavy monsoons. Research conducted by the Brazilian Technical Commission for Agricultural Loss Reduction revealed that storage losses for rice in Brazil stand at 2.4% [87]. Chowdhury et al. [88] found that farmers in Bangladesh incurred losses of roughly 2.33% due to issues with their storage facilities and containers. In addition, Costa [11] also estimated substantial losses of up to 59.48% in maize grains following a 90-day storage period in conventional structures such as granaries or polypropylene bags.

The design of the storage structure plays a significant role in keeping agricultural products safe. Across numerous developing nations, especially in South Asia and Africa, storage facilities include granaries (crafted from locally sourced materials like straw, bamboo, and bricks), mud bins, earthen pots, Bokhari, Kanaja, Kothi, Gummi, plastic containers, and plastic/steel drums, among various others [89]. Hermetic/airtight containers (plastic) are also utilized due to their ease of installation, minimal infrastructure requirements, and cost-effectiveness [90]. Hermetic storage has proven highly effective in minimizing losses for long-distance shipment/international trade or long-term storage. The FAO-developed metal silos have gained significant popularity in Kenya and Mozambique for their nearly complete elimination of insect-related losses [91]. These silos are suitable and cost-effective for small-scale storage [92,93,94].

An on-farm permanent storage system can still be relatively costly. For example, grain silos, which have large capacities, are expensive, but they are commonly used at public and commercial levels, where losses are minimal. A recently developed alternative storage system involves the use of grain harvest bags, often referred to as “Cocoons”. Developed in Argentina, these harvest bags (silo bags) are membrane-based storage units. Roughly 45 million tons of grain are stored in silo bags annually in Argentina [95]. The principle behind these bags is that gas-tight grain respiration leads to the production of CO_2_ and the depletion of O_2_, effectively suppressing the activity of any fungi or insects present. It has been reported that in Australia, grain harvest bags allow farmers to manage their grain harvest more effectively and maintain the grain in a safe condition for many months.

#### Minimizing Storage Loss

A well-designed storage system has the potential to minimize PHL. Several studies have highlighted that the highest incidence of PHL occurs during the storage phase, often due to inadequate or ineffective storage infrastructure. Donate and de Pablo [96] highlighted the importance of disseminating knowledge about enhanced storage structures and management practices, which has the potential to result in a significant decrease in food wastage, possibly up to 98%.

Addressing abiotic factors, particularly moisture levels, and weather conditions, is also pivotal in mitigating storage losses. Agricultural products are susceptible to moisture, necessitating proper drying before storage to maintain a safe moisture level. Hence, it is advised to keep the moisture level below 13% for long-term storage and below 15% for storage periods of less than six months [97] (Table 7). Moreover, relocating grain from smallholder farms to modern, well-regulated centralized storage facilities is an effective method for minimizing storage losses [98].

### 8.8. Milling and Processing

Milling or processing cereal grains involves using mechanical means to remove their outer skin and hull [106]. However, it is common for both the quantity (weight) and quality (value, micronutrient content) to be reduced during milling and processing [107]. For example, when rice is polished or husked, the bran and germ, containing essential elements like zinc, iron, vitamins, calcium, phytate, and some proteins, are removed [108,109]. Oghbaei and Prakash [110] demonstrated that rice and wheat experienced a 69% and 67% reduction in their iron content, respectively, as well as a 39% and 73% reduction in their zinc content, respectively, during processing.

The milling process uses machines categorized into traditional and modern types. Conventional milling frequently leads to a significant proportion of broken grains and a loss of valuable rice bran mixed with husks. In contrast, modern rice mills offer advantages such as minimal losses (5~30%), the ability to grade the rice, and separate valuable byproducts like rice bran and husks [111].

The yield from milling, particularly for rice, is profoundly impacted by elements like the moisture content of the grain and the degree of polishing. Grain harvested with high moisture levels should be promptly dried to 14% within 24 h to ensure secure storage and milling. Rumandla et al. [112] observed that milling efficiency is negatively impacted when the paddy is not thoroughly cleaned. For instance, in Bangladesh, milling loss was recorded at 3.78%, while in Nepal and Indonesia, it was 4.4%. On the other hand, over-hulling is a significant issue in South Asian countries, particularly Bangladesh and India. Excessively polishing of the grain can lead to weight loss, nutritional deficiency, reduced shelf life, and increased vulnerability to insect pests [113]. Sheahan and Barrett [16] point out that overly processed cereals (over-polished) have lower levels of vitamins, minerals, and beneficial oils.

#### Minimize Milling Loss

To minimize milling losses, it is advisable to utilize modern milling machinery that allows for separate husking and polishing processes. Automated processing mills are beneficial in regulating the polishing procedure, leading to increased milling yields through loss reduction [114]. This approach enhances the overall product quality by minimizing breakage and ensuring a uniform polishing effect. To achieve the most effective processing outcomes, it is crucial that the grain is sufficiently dry, with a moisture content of less than 14%, and thoroughly cleaned before milling.

### 8.9. Bagging, Packaging, and Labelling

Packaging is influential in meeting the market demand for aged grain or seeds [115]. It is intricately linked with processes like labelling and branding, especially for grain exports. Opting for lower-quality packaging can result in increased PHL because of contamination, insect infestation, and a decline in commercial value. The recommended standard for packaging involves using new, clean, and dry materials such as poly-woven bags, high molecular, high-density polyethylene paper packages, or other food-grade plastic/packaging materials. To ensure high quality, graded rice, wheat, and maize should be stored in top-notch bags with appropriate labelling. Each container should be securely closed and properly sealed. Recent research has also delved into developing and utilizing biodegradable packaging materials, like plastics derived from sugar cane [116]. These cost-effective bags not only help harness the value of sugar cane but also contribute to environmental conservation.

## 9. Off-Farm Activities: Effect of Non-Technological Factors

Off-farm or indirect actors in the grain supply chain encompass elements like transportation links (roads, rail), market facilities, warehouses (integral parts of food marketing infrastructure), and investors. In Karnataka, India, around 75% of total PHL arises at the farm level, while the remaining 25% occurs at the marketplace level [117]. Among these indirect factors, establishing warehouses presents a significant opportunity for reducing PHL. Additionally, Fauziana et al. [118] propose that improvements in infrastructure, the establishment of warehouses, the development of rural markets, and efficient supply chain strategies are pivotal in achieving PHL reduction. Notably, initiatives in developing countries to modernize the grain value chain within the agricultural sector hold considerable importance.

Kiaya [14] shows that marketing is a vital component of the agricultural production chain. Moreover, robust financial markets should grant smallholders access to credit, savings, and insurance, enabling them to invest in reducing postharvest losses. Numerous developing or less developed countries have an insufficient postharvest system and a fragile financial infrastructure, leaving producers’ livelihoods susceptible. Conversely, warehouses play a critical role in the marketing system and can streamline market exchanges by reducing transaction costs [119]. In Australia, storage and milling infrastructure are typically integrated into or located near regional towns, creating marketing opportunities that align with the principles of Product, Price, Promotion, and Place (4P principles) [120].

## 10. Postharvest Loss Mitigation: Systematic Intervention, Approaches, and Best Practices

Overall, eliminating postharvest loss may be impossible, but achieving a 50% reduction is feasible and beneficial [54]. Efficient postharvest management on the farm and throughout the supply chain is crucial in mitigating PHL. Various strategic options and promising technologies are available and currently in use to minimize product losses and ensure grain quality in the postharvest chain [121] (Table 8).

Handling postharvest operations is immensely complex due to product diversity, variations in crop physiological status, and climatic disparities [122]. Clark and Hobbs [123] advocate for national and international research organizations, agricultural extension departments, Non-Government Organizations (NGOs), and technology manufacturers to collectively advance efforts to manage and reduce postharvest losses effectively. Selecting the right technology package is also greatly important. Research highlights that this choice should be tailored to factors such as production scale, crop variety, weather conditions, and farmers’ capability and willingness to invest. For developing countries, prioritizing appropriate technology over advanced technology is crucial (Table 9).

## 11. Overall Strategies and Effects

### 11.1. Global Initiative and Postharvest Loss Reduction

The World Food Program is engaged in initiatives related to storage technology in Uganda and Burkina Faso [11]. The FAO has also made efforts to promote the adoption of metal silos in African countries [133]. The International Rice Research Institute (IRRI) has worked on extending combine harvester and drying technology in Vietnam [39]. Other initiatives, such as “SAVE FOOD”, “FUSIONS”, “WRAP UK”, “OECD Food Chain Analysis Network”, “Global Food Banking Network”, and the “Think. Eat. Save-Campaigns” have also played significant roles.

### 11.2. Postharvest Loss Reduction and Impact on Society

Postharvest losses have far-reaching implications, affecting food security, economies, societies, and the environment. Reducing these losses makes more food available to farmers, benefiting impoverished individuals in rural and urban settings. Kiaya [14] highlights that this reduction lowers prices and improves food security. Chapagain and Raizada [134] emphasize that small-scale farmers in developing countries, frequently on the brink of food insecurity, could experience a significant and immediate improvement in their livelihoods with reduced food losses. An estimated calculation of food savings and economic value in developing countries is presented in Table 10.

### 11.3. Value of Postharvest Research

The principal objective of postharvest technology research is to minimize losses in both the quality and quantity of produce [14]. There is a need for more well-established postharvest (PH) research networks outside of developed regions, and global funding mechanisms that can support interdisciplinary collaborations need to be established [136]. Kitinoja and Barrett [137] highlight that less than 5% of agricultural research funding is allocated to the postharvest research sector. This situation should be changed. Additionally, it is crucial to motivate growers to adopt postharvest technology that offers a high return on investment and cost–benefit ratio, even if, in some cases, the implementation of PH technology may be demanding. Embracing an interdisciplinary research and development approach is essential to address these imperatives, and this is expected to remain valid in the future.

## 12. Advanced Technologies and Practices to Reduce PHL

Munarso and Widayanti [138] reported that one-third of food produced is lost or wasted between harvesting and consumption processes. To combat this, various organizations and researchers have conducted advanced research, which includes smart and precision postharvest practices, IoT applications for harvesting systems, novel drying [139] and storage (Bubble and Tunnel), active and smart packaging, cool chain processes, coating technologies, etc. (Table 11). Scientists worldwide are also actively researching other safer and more sustainable fumigation compounds to replace or rotate with phosphine to help manage insect/pest issues in cereal grains [140]. Computer models are increasingly used to study the behavior of grain storage ecosystems [141]. Computer simulation is also an effective and relatively inexpensive method for optimizing fumigation practices and storage design.

New packaging with built-in smarts can also reduce PHL. Degradable bioplastic and nano cellulose-based packaging are environmentally friendly innovations that might help mitigate food quality and quantity losses [142]. These high-tech systems use sensors or indicators to keep tabs on a product’s condition in real time [143]. For example, a box can be made from recycled cardboard that will change color to indicate when the fruit needs to be eaten. This not only helps avoid food waste by ensuring freshness but also extends the shelf life of groceries. By using recycled materials in this smart packaging, companies are tackling environmental concerns, reducing waste, and saving resources [144]. These innovations show a commitment to sustainability and the smart use of materials, creating a system where things are constantly reused and recycled [145]. As a result, smart packaging with recycled materials will be able to set new industry standards in the future. This is a win–win for functionality and the environment.

**Table 11:** Published articles on advanced technologies and practices.

Advanced Postharvest Technology in Practice	Purposes	Examples	References
Harvesting	IoT-based harvesting system	Combine harvester technology that is operated and controlled through apps in the farmer’s hand	[146]
Cold handling and storage	Thermal and non-thermal processing of harvested agricultural products to reduce spoilage and enhance shelf life	Storing grain in a silo, specially made from galvanized steel, is identified as the most preferred storage technique	[147]
	Advanced with innovations such as aeration, refrigerated storage, modified atmospheric storage, and hermetic storage systems	High-pressure processing, cold plasma, UV radiation, and gamma irradiation are examples of green technologies used for agricultural commodities	[148]
	Refrigerator-based cold storage systems to prevent perishable food losses and improve shelf life	Reducing the food loss of fruits and vegetables	[149]
Drying and modelling thereof	Quality and quantity improvement of crops as well as low operating cost	Tunnel dryer, spray drying, drum dryer, freeze-drying, microwave drying, and fluidized bed drying with various combinations of methods, including hybrid drying, superheated steam drying, reflectance window, impingement drying, high electric field drying, or electrode hydrodynamic drying	[150,151]
Novel packaging or intelligent packaging and recycled materials	Extend shelf life, monitor freshness, display information on quality, and improve safety	Smart packaging technologies include data carriers, indicators, and sensors	[152]
	UV absorbers can reduce food waste and optimize customer satisfaction	GrainPro and PICS bags reduce pest intensification and quality	[153]
Supply chain and value chain management/optimization modelling	Monitoring, grading, and classification, predicting quality properties, and forecasting chemical, physical, and nutrient characteristics	Supply/value chain modelling helps E-commerce	[154,155,156]
Track and trace systems	Movement of product, product data, producer information, supply chain and consumer information	AI technology for sensing data collection and analyses and determination of the contamination system of food	[157,158]
Emerging transport technologies as well as modelling and simulation	Optimum logistics for grain transportation and infrastructure to minimize the total system cost	Optimum Rake Allocation Algorithm model and GIS-based transport system for commercial products	[159,160,161]

## 13. Challenges and Outlook of Postharvest Loss (PHL) Reduction

The postharvest loss (PHL) of grain poses a significant concern for achieving global food security and sustainability. These losses impose a substantial economic burden and contribute to food insecurity. Various postharvest technologies and knowledge have been developed to minimize both the quality and quantity of grain postharvest loss. However, numerous challenges contribute to the persistently high rates of grain PHL, particularly in developing countries. These challenges include (1) inadequate infrastructure, drying, and storage facilities; (2) poor handling practices; (3) limited access to knowledge and technology; and (4) a lack of financial resources. Therefore, future development and research in postharvest technology should be focused on aspects such as research, technology development, effective applications, information dissemination, and policy issues.

The recent progress and advancements in modern technology can further open possibilities for applying postharvest technology and information, offering positive prospects for ensuring food security. Particularly, recent studies include (i) prioritizing research and development efforts towards the development of cost-effective and accessible drying/storage technologies incorporating automation, IoT, and sensor-based systems; (ii) implementing educational programs, extension services, and training to enhance effective harvesting, drying, and transportation practices, alongside best practice campaigns; (iii) leveraging access to knowledge and technology to provide farmers with timely and relevant information on strategies for PHL reduction; (iv) governments and development organizations offering financial assistance to smallholder farmers to invest in PHL reduction measures; (v) enhancing market linkages to motivate farmers to invest in PHL reduction actions by ensuring fair prices for their produce; and (vi) advocating for policies that support PHL reduction efforts, including subsidies for postharvest technologies and tax incentives for adopting improved handling practices.

Overall, strengthening institutions engaged in research, extension services, and regulatory oversight represents a significant step toward creating an enabling environment for effective postharvest management and could be a step forward to food security.

## 14. Conclusions

This comprehensive review highlights the substantial variability in postharvest losses (PHLs) observed on a global scale. The PHLs vary widely worldwide due to many factors, including crop type, farming methods, weather, and economic differences. The extent of losses in developing countries is particularly concerning, which can surge to an alarming 30~40% of total production. Notably, low-income countries are highly affected by grain losses in the initial and intermediate stages of the grain supply chain. In contrast, industrialized nations also have significant waste, but it is predominantly at the consumption stage.

The thorough examination presented in this paper provides a compelling summary of the effectiveness of an array of postharvest technologies, the intricate dynamics of the supply chain, nation-specific data, and the overarching panorama of PHL. Within this supply chain framework, drying losses often manifest at the farm level, while storage losses tend to be more pronounced. The substantial incidence of PHL in developing countries, particularly in the case of rice, is predominantly attributed to the use of outdated machinery and methods. Maize and wheat exhibit lower susceptibility to losses at the field level but have higher storage losses. Interventions involving technological advancements and embracing best practices are pivotal to mitigating PHL.

Several promising on-farm practices and cutting-edge technology, ranging from advanced harvesters and threshers to automated milling and innovative drying and storage techniques, can positively impact the industry. Future grain storage technology will also become more intelligent and environmentally friendly. Knowledge transfer and active farmer and community engagement to overcome the adoption barriers are critical, particularly in developing countries.

## Figures and Tables

**Table 1:** Moisture content and main losses in different PH operations of field crops (rice).

Operation	Desired Moisture Content (MC)	Primary Losses
Harvesting	20–25%	Shattering if the grain is too dry
Threshing	20–25% for mechanical threshing <20% for hand threshing	Incomplete threshing Grain damage and cracking/breakage
Drying	<14%	Spoilage, fungal damage Discoloration
Storage	<14% for grain storage <13% for seed storage <9% for long-term seed preservation	Fungal, insect, and rat damage Loss of vigor
Milling	14%	Grain cracking and breakage Over-milling

**Table 3:** Published data on postharvest losses in various crops in some non-major rice-growing countries.

Country	Significant Findings
Paraguay	Horticultural products: 8~15%; strawberries: 12%; cereals and oilseeds: about 5%
Haiti	Loss of essential food crops (vegetables, fruits, tubers, cereals and legumes): 35%
Serbian	Wheat: 0.5~1%; Corn: 0.5% at field level Wheat and corn: 1~2% stored on the farm
Peru	Corn, beans, and wheat: 10 to 15%
Tajikistan	Wheat: 1.5%; corn: up to 4%
Guatemala	Cereals: 15%
El Salvador	White corn and red beans: 8%; Rice and sorghum: 6%
Nicaragua	Corn, beans, and rice: at least 15%
Panama	Wheat: 20%; beans: 12~18% at storage level
Ecuador	Corn: 20%
Ethiopia	Sorghum: 11.6%; Wheat: 9.9%; Maize: 16.8%
Serbian	Wheat: 1.5%; Corn: 4%
Tajikistan	Cereals: 15%
Nicaragua	Corn, beans, and rice: at least 15%
Panama	Corn and beans: 20%; Tuber and Cassava: 12–26%

Source: https://2009-2017.state.gov/documents/organization/220958.pdf (Postharvest Loss Challenges, Discussion Paper; accessed on 21 April 2024).

**Table 4:** Causes and responsible factors for postharvest losses in the grain supply chain.

Steps	Causes	Factors toward Losses	Loss Components
On-farm interventions			
Harvesting and handling at harvesting	Lack of mechanization Lack of timely action Susceptible variety	Genetic traits, susceptible to loss Timing of harvest not optimal Shattering loss during and due to delayed harvesting Insects, rodents, and birds attack standing crops Broken grains Edible crop left in the field Mechanical shattering	G (Shattering, losing, standing crop loss)
Threshing	Immature or over-mature crop harvesting Poor technique Unsuitable machinery	Improper separation of grains Broken grains due to threshing Scattering of grains Improperly cleaned grains yielding high losses during storage and milling	G + V (Separation, scatter, threshing loss)
Drying	Depending on sun drying Bad weather condition Drying is delayed Lack of dryer	Birds and rodents attack crops lying in the field Contamination with foreign materials Scattering of grains Contamination by spoilage, fungi, and bacteria Fissuring of grains due to overheating in the sun	G (Scatter, drying loss)
Storage	Inadequate storage facilities Improper clean grains	Insect infestation Aflatoxin and mycotoxins developed Discoloration Natural drying out of food	G + V (Storage loss)
Processing, cleaning, grading, hulling, pounding, grinding, soaking, sieving, milling	Lack of machinery/technology Poor handling facilities	High level of broken grains Spillage in traditional milling/husking Contamination with foreign materials Process losses High milling losses due to incomplete or over-drying	G + V (Weight, value, and mixing loss)
Packaging and labeling	Lack of machinery Poor technique	▪ Inappropriate packaging ▪ Grain spillage from sacks: attacks by pests	G + V (Weight and Value loss)
Transportation	Poor road conditions and connections Inadequate facilities	▪ Quality loss due to bruises ▪ Spillage of grains ▪ Contamination if transported in bulk in uncovered vehicles ▪ Damage during transport	G + V (Weight, value, and mixing loss)
**Off-farm interventions**			
Rural market, selling, distribution	Government strategies	Inadequate market structure and policies Lack of information Lack of capital and resources Credit constraints	V
Infrastructure		Lack of investment in roads, market facilities, electricity, etc. Lack of networks among roads, railways, and sea shipment	V
Warehouse receipt system		Lack of investment in warehouses Lack of private investment and initiatives Poor storage/stock management	V
Efficient value chain		Lack of interaction between retailers, wholesalers, and restaurants Inability to transmit information Absence of monitoring	V

Note: G is grain loss, and V is value loss. Source: Parfitt et al. [12] and authors.

**Table 5:** Overview of tools used in grain harvesting system.

Harvesting System		Cutting Equipment	Hauling/ Moving	Threshing	Cleaning
Manual system	Manual harvesting and threshing by beating	Cutting with a sickle	Carrying crop by hand	Hand threshing	Winnowing or grain cleaner
	Manual harvesting and threshing by pedal thresher			Pedal thresher	
	Manual harvesting and threshing by trampling			Animal trampling	
Manual harvesting with machine threshing		Cutting with a sickle	Collecting and hauling crops by hand	Feed-in thresher	Winnowing, thresher cleaner, or grain cleaner
Machine reaping with machine threshing		Reaper	Hauling crop by hand	Feed-in thresher	Winnowing, thresher cleaner, or grain cleaner
Combine harvest		Combine machine (five activities at a time: cutting, moving, threshing, cleaning, and bagging)			

**Table 6:** Safe moisture content for storage.

Storage Period	Required Moisture Content for Safe Storage	Potential Problems
2 to 3 weeks	14~18%	Mold, discoloration, respiration loss
8 to 12 months	12~13%	Insect damage
More than 1 year	9% or less	Loss of viability

Source: [75].

**Table 7:** Management and technology for storage loss reduction.

Practice	Technique/Technology		Significant Findings	References
Management	Moisture content	Below 13%	✓ Long-term storage	[97]
		Less than 15%	✓ Short, less than 6 months	
		Above 16%	✓ Few weeks	
	Aeration	Airflow 2–20 L/s per tonne	✓ Bulk storage	[99]
	Chemical fumigation	▪ Methyl bromide ▪ Phosphine	✓ Commonly used in developing countries ✓ Control *enormous grain borer* insects for cereal crops	[100]
		▪ Actellic Super	✓ Frequently used in Africa (Kenya/Tanzania)	
		▪ Nitrogen or ethyl bromide	✓ Safe for humans and environment	[101]
	Natural insecticides	▪ Chenopodiaceae-leaves/herbs	✓ Suitable for smallholders	[102]
		▪ Crude palm kernel oil ▪ Crude rice bran oils ▪ Pure soybean oil ▪ Crude cottonseed oils	✓ Effective for grain weevils and borers ✓ Expensive, not suitable for commercial use	
Storage structure/system	Hermetic system	▪ Plastic bag ▪ Super Grain ▪ Purdue (PICS) bags	✓ Commonly used in smallholders ✓ PHL less than 1%	[92,100]
	Silo	▪ FAO metallic silo ▪ Local metallic silo	✓ Commonly used in Asian and African countries ✓ Affordable cost	[103]
	Plastic/steel drum	▪ Small to medium farm	✓ Commonly used in South Asia ✓ Cost-effective and easy to handle	[104]
	Grain harvest bag	▪ Medium- to large-scale farm	✓ Commonly used in developed countries ✓ Medium- and large-scale (shipment) use	[105]

**Table 8:** Postharvest interventions and technologies with grain production and supply chain.

Stage	Intervention	Technology	Information
Pre-harvest	▪ Breeding for resistance to shattering ▪ Resistant to insect, pest, and disease susceptibility	-	▪ Research and development
Proper harvesting	▪ Mechanization for farmers’ groups, stakeholders, and entrepreneurs	▪ Reaper ▪ Reaper-Binder ▪ Combine harvester ▪ Mechanical/power intervention	▪ Grain moisture (20~25% for rice) ▪ Straw/plant stem and grain color ▪ Hard doe/maturity stage ▪ Days after flowering ▪ Harvesting quicker
Careful transportation from the field and along the supply chain	▪ Mechanical system	▪ Power transport system ▪ Covered van	▪ Aware of weather conditions ▪ Planning with a view to distance
Threshing and Shelling (including proper equipment)	▪ Mechanized thresher/Sheller	▪ Open drum thresher ▪ Closed drum thresher/Sheller ▪ Head feed thresher ▪ Combine harvester	▪ Threshing quicker ▪ Information about seed and non-seed ▪ Awareness of admixture
Proper drying	▪ Solarization and improved dryer ▪ Mechanical dryer	▪ Judicial sun drying ▪ Flatbed dryer ▪ Thin layer dryer ▪ Recirculating dryer ▪ Nobel drying technology	▪ Information about seed and non-seed ▪ To minimize moisture content ▪ Monitoring grain humidity during drying to avoid mold growth
Cleaning	▪ Mechanical system	▪ Winnower ▪ Power cleaning system	▪ Information about grain (seed and consumption) ▪ Purpose of use
Sorting and grading	▪ Mechanical system	▪ Grader	▪ Information about seed and consumption ▪ Concern about admixture
Storage	▪ Protected storage structures ▪ Improved pest/fungi management ▪ Safe chemical use and familiarity with their use	▪ Hermetic storage system ▪ Metal silo ▪ Careful use of grain protector ▪ Knowledge of protector use ▪ Integrated pest management system	▪ Duration of storage ▪ Aim of local use or export ▪ Concern regarding expiry recommendations, adulteration ▪ Careful grain loading and stacking
Packaging and branding	▪ Update technology ▪ Value-added system	▪ Hermetic bag ▪ Plastic laminated bag ▪ Labeling of needed information	▪ Purpose of storage ▪ Aim of local use or export ▪ Insect control and prevention ▪ Value-added activities
Access to market information	▪ Institutional arrangements for grain marketing ▪ Accessing market information and understanding of seasonal price fluctuations	▪ Facilitate access to market information. ▪ Inventory credit means of offering stocks of cereals as guarantees for cash loans	▪ Understanding of household food budgeting requirements
Communication and learning regarded PHL issues	▪ Media, extension, and education through different methods ▪ PH-related issues ▪ Promote learning alliances	▪ Training the trainers ▪ Farmer field schools ▪ Education curricula	▪ Action-oriented training ▪ E-learning system ▪ Platform to engage with public & private stakeholders
Facilitating factors	▪ Research and development	▪ Resistant varieties ▪ Cost-effective drying methods ▪ Investments in infrastructure (roads, storage, etc.) ▪ Business model for PHT	▪ Integrating postharvest loss reduction into agricultural policies ▪ Facilitating credit to smallholders and other chain actors ▪ Enhancing postharvest capacities of service providers and extension services

**Table 9:** Recommended postharvest technology used and its impact.

PH Intervention	Technology	Impact	Result **	References
Grain varieties with better PH traits	No variety yet available	Maintain resistance quality to insect infestation Low shattering tendency	✓ Reduce physical and quality PHL	[90]
Transport	Covered van Two-wheel tractor Truck	Reduce physical PHL	✓ Increased quantity and better quality	[124]
Better harvesting	Reaper Combine harvester	Decreases the labor involvement Timely harvesting and processing Reduces trouble and cost	✓ Reduce physical PHL ✓ Better quality grain ✓ Reduce drudgery	[125,126]
Threshing/Shelling/Winnowing	Mechanical thresher/sheller Winnower	Reduces labor requirements Timely harvesting and processing	✓ Reduce physical PHL ✓ Better quality grain	[127]
Drying	SSR low-cost dryer Flatbed dryer Re-circulating dryer Nobel drying technology	Less physical deterioration Protected from mycotoxin and aflatoxin	✓ Better quality grain ✓ Good market value	[128]
Storage/warehouse	Metal/plastic silo IRRI superbag Hermetic system	Convenient for long-term preservation Protects from deterioration by pests and insects	✓ Better quality grain will be available for consumption or to sell ✓ Increase market value	[129,130]
Milling	Engleburg rice mill Semi-auto rice mill Auto rice mill Hammer/flour mill	High milling yield	✓ Better quality grain ✓ Better market value	[131]
Collective marketing and credit system	Cooperative system Organizational lone	Increased buying capacity Enhanced production capability Highest market price	✓ Improve the livelihood from grain production ✓ Upgrade the technology	[132]

** The ultimate impact pathway will be to decrease poverty; increase food and nutrition security; improve health and sustainability; enhance gender equity; increase profitability for men and women; other value chain actors; ensure high-quality grain; and enrich the livelihoods of farmers.

**Table 10:** Estimated economic values of postharvest losses in developing countries, 2021-2022.

Description	Rice		Maize		Wheat		Barley		Sorghum	
	Estimate	Mt	Estimate	Mt	Estimate	Mt	Estimate	Mt	Estimate	Mt
World production	100%	700	100%	1100	100%	735	100%	140	100%	60
Production in developing countries	90%	630	70%	770	50%	367.5	50%	70	50%	30
World production of small- and medium-scale farmers (assumed to be in developing countries)	80%	504	60%	462	80%	294	80%	56	80%	24
Total postharvest losses	14%	70.56	11%	50.82	12%	35.28	10%	5.6	10%	2.4
Technological interventions reduce PHL	5%	25.2	8%	36.96	8%	23.52	7%	1.96	7%	0.84
Total economic savings	250 *	63 **	200 *	73.92 **	300 *	70.56 **	500 *	9.8 **	500 *	4.2 **

Note: * Assuming the cost (USD/ton), ** USD billion. Source: Author calculation; Food Balance Sheet Data, 2020–2021 (https://www.fao.org/faostat/en/#data; accessed on 21 April 2024); and [24,135].

## Data Availability

No new data were created or analyzed in this study. Data sharing is not applicable to this article.
